# Clinical and sociodemographic correlates of suicidality in patients with major depressive disorder from six Asian countries

**DOI:** 10.1186/1471-244X-14-37

**Published:** 2014-02-13

**Authors:** Ah-Young Lim, Ah-Rong Lee, Ahmad Hatim, Si Tian-Mei, Chia-Yih Liu, Hong Jin Jeon, Pichet Udomratn, Dianne Bautista, Edwin Chan, Shen-Ing Liu, Hong Choon Chua, Jin Pyo Hong

**Affiliations:** 1 Department of Psychiatry, Asan Medical Center, Ulsan University College of Medicine, Seoul, South Korea; 2 Department of Psychological Medicine, Faculty of Medicine, University of Malaya, Kuala Lumpur, Malaysia; 3 Peking University Institute of Mental Health, Beijing, China; 4 Department of Psychiatry, Chang Gung Medical Center and Chang Gung University, Tao-Yuan County, Taiwan; 5 Department of Psychiatry, Depression Center, Samsung Medical Center, Sungkyunkwan University School of Medicine, Seoul, South Korea; 6 Department of Psychiatry, Faculty of Medicine, Prince of Songkla University, Songkhla, Thailand; 7 Singapore Clinical Research Institute, Singapore, Singapore; 8 Duke-National University of Singapore, Singapore, Singapore; 9 Department of Psychiatry, Mackay Memorial Hospital, New Taipei City, Taiwan; 10 Institute of Mental Health, Woodbridge Hospital, Singapore, Singapore

**Keywords:** Suicide, Major depressive disorder, Risk factor, Social support

## Abstract

**Background:**

East Asian countries have high suicide rates. However, little is known about clinical and sociodemographic factors associated with suicidality in Asian populations. The aim of this study was to evaluate the factors associated with suicidality in patients with major depressive disorder (MDD) from six Asian countries.

**Methods:**

The study cohort consisted of 547 outpatients with MDD. Patients presented to study sites in China (n = 114), South Korea (n = 101), Malaysia (n = 90), Singapore (n = 40), Thailand (n = 103), and Taiwan (n = 99). All patients completed the Mini-International Neuropsychiatric Interview (MINI), the Montgomery–Asberg Depression Rating Scale (MADRS), the Global Severity Index(SCL-90R), the Fatigue Severity Scale, the 36-item short-form health survey, the Sheehan Disability Scale, and the Multidimensional Scale of Perceived Social Support (MSPSS). Patients were classified as showing high suicidality if they scored ≥6 on the MINI suicidality module. Multivariate logistic regression analysis was used to examine sociodemographic and clinical factors related to high suicidality.

**Results:**

One hundred and twenty-five patients were classed as high suicidality. Unemployed status (adjusted odds ratio [OR] 2.43, p < 0.01), MADRS score (adjusted OR 1.08), p < 0.001, and GSI (SCL-90R) score (adjusted OR 1.06, p < 0.01) were positively related to high suicidality. Hindu (adjusted OR 0.09, p < 0.05) or Muslim (adjusted OR 0.21, p < 0.001) religion and MSPSS score (adjusted OR 0.82, p < 0.05) were protective against high suicidality.

**Conclusions:**

A variety of sociodemographic and clinical factors were associated with high suicidality in Asian patients with MDD. These factors may facilitate the identification of MDD patients at risk of suicide.

## Background

It is estimated that approximately 1 million people worldwide commit suicide annually, and about 60% of these people are from Asian countries [1,2]. The suicide rate in East Asian countries, including South Korea, Japan, and China, is especially high. According to a 2011 report from the World Health Organization’s worldwide initiative for the prevention of suicide, Korea ranked third, Japan ranked ninth, and China ranked twenty-fourth out of 105 countries for suicide rate [3]. Despite such compelling figures, suicide is relatively under-researched, and preventive approaches in Asian countries are limited compared to those in European and American countries [4-6].

Studies have consistently reported that major depressive disorder (MDD) is closely related to suicide, suicidal ideation, suicide planning, and suicide attempts and is a significant risk factor for suicide [7,8]. According to previous studies, severe or extended depression [9-12], advanced age [9], low level of education [13], low level of social support and occupational functioning [9,11], lack of a partner [11,12], current alcohol dependence or substance abuse [9,11,13], negative life events [14], and impulsivity and hostility [10,15,16] have all been reported to be risk factors for suicide attempts in MDD. However, the etiology of MDD is extremely complicated, and the generalization of suicide risk factors is difficult because of differences between studies in the populations studied and the methods employed. In particular, the profiles of risk and protective factors of suicide in Asian countries differ from those of Western countries [10].

Recently, we reported that melancholic features and hostility were associated with suicidal risk in MDD patients from six Asian countries [17]. However, the cited study mainly focused on melancholic features and did not examine important factors such as religion, functional impairment, and poor social support. Interestingly, the prevalence of MDD is lower in East Asian countries than in European and American countries, but suicide rates are higher [18,19]. This suggests that in East Asian countries, various clinical, social, and cultural factors, including religious practices, may be related to suicide in addition to psychiatric disorders such as MDD.

Although several studies have provided information on the risk factors for suicide in Asian countries [20-22], comprehensive examination on the characteristics of suicide in MDD by multi-country comparative analysis was few. Accordingly, the aim of the present study was to evaluate the sociodemographic and clinical factors related to suicidality in MDD patients from six Asian countries (China, South Korea, Malaysia, Singapore, Taiwan, and Thailand).

## Methods

### Study design and settings

This study uses data from the Study on the Aspects of Asian Depression (SAAD) [20]. The participants and method of the present study are the same as those of the Recognizing Ethnic Differences in Depression (REDD) study [17], a multi-country, cross-sectional, observational study of depression in clinical settings carried out during 2008–2011. Thirteen study sites were established across six Asian countries: China, South Korea, Malaysia, Singapore, Taiwan, and Thailand. The study sites were as follows: Beijing Anding Hospital (Beijing, China), Institute of Mental Health (Beijing, China), Shanghai Mental Health Center (Shanghai, China), Samsung Medical Center (Seoul, Korea), Asan Medical Center (Seoul, Korea), Kyungpook National University Hospital (Daegu, Korea), Inha University Hospital (Incheon, Korea), University of Malaya Medical Center (Kuala Lumpur, Malaysia), Institute of Mental Health Woodbridge Hospital (Singapore), Chung Gang Memorial Hospital (Taoyan county, Taiwan), McKay Memorial Hospital (Taipei City, Taiwan), Maharaj Nakorn Chiang Mai Hospital (Chiang Mai, Thailand), and Prince of Songkla University (Songkla, Thailand). All study sites provided psychiatric care for the public or private sector. The study was approved by the Institutional Review Board or Ethics Committee of Asan Medical Center and each respective site.

### Participants

Participants were prospectively enrolled in the study and were recruited from outpatients who were seeking psychiatric treatment at a study site. Individuals presenting for an intake appointment were approached by a study coordinator and informed about the study. After the study details had been fully explained, written informed consent was obtained from each participant. The inclusion criteria were as follows: i) age 18–65 years; ii) a positive response (“yes”) to the Mini-International Neuropsychiatric Interview (MINI) [21] question A1 (depressed mood) and/or A2 (loss of interest); and iii) a diagnosis of MDD according to the DSM-IV criteria [22] that was assessed by the MINI. The exclusion criteria were as follows: i) unstable medical condition; ii) mood disorder due to medical conditions and/or substance abuse; iii) psychotic or bipolar disorder; iv) clinically significant cognitive impairment; v) treatment with psychotropic medication within the previous month; vi) treatment with a benzodiazepine within the previous week; and vii) treatment with a long-acting antipsychotic medication within the previous 3 months. All other psychiatric and comorbid conditions were permitted.

The following sociodemographic characteristics were recorded: age, sex, marital status (married or co-habiting; widowed or divorced; never married), work status (employed; homemaker or student; unemployed), and education (none or primary; secondary or vocational; college). The following clinical characteristics were recorded: age at first onset, length of illness, number of past psychiatric hospitalizations, and depression severity.

### Assessment

Participants completed several self-report questionnaires in the presence of the study coordinator. A face-to-face diagnostic evaluation was then conducted with the site investigator before the participant met with their treating clinician. Data collection was accomplished in a single visit. Suicidality is the likelihood of an individual completing suicide and include suicidal ideation, self-injurious behavior, suicide attempts, and suicide despite their very different consequences for the patient. In the present study, the term “suicidality” includes the full spectrum of suicidal thoughts (thoughts about wanting to be dead) and suicidal acts (previous self-destructive behaviors [23] with at least some intent to end one’s life), in keeping with a previous study [23].

Suicidal ideation and behaviors were assessed with the MINI suicidality module [21]. The MINI suicidality module was used to rate the risk of suicide. The module comprises 6 questions about suicidal ideation and behavior: (1–5) In the past month, did you 1. think you would be better off dead or wish you were dead? (1 point), 2. want to harm yourself? (2 points), 3. think about suicide? (6 points), 4. have a suicide plan? (10 points), 5. attempt suicide? (10 points). 6. In your life, have you ever made a suicide attempt? (4 points). The total number of points is used to classify the current suicide risk on three levels. Scores ranging from 1 to 5 are considered low risk, from 6 to 9 are moderate, and above 10 are high. According to the previous study investigating predictive value of MINI suicidality module, the sensitivity and specificity for suicide attempts after 12 months in patients with moderate-risk MINI sum scores are 0.73 and 0.62, and with high-risk, the MINI sum scores are 0.61 and 0.75[24]. The positive and negative likelihood ratios for patients with moderate-risk sum scores are 1.9 (95% CI, 1.1-3.2) and 0.44 (95% CI, 0.26-0.74), respectively, and in patients with high-risk sum scores, they are 2.4 (95% CI, 1.9-3.0) and 0.52 (95% CI, 0.42-0.65) [24]. In this study, depression severity was assessed with the Montgomery-Asberg Depression Rating Scale (MADRS) [25], psychiatric symptoms were assessed with the Global Severity Index(GSI provided by SCL-90-R) [26], fatigue severity was assessed with the Fatigue Severity Scale (FSS) [27], health-related quality of life was assessed with the 36 item short form health survey (SF-36) [28], disability was assessed with the Sheehan Disability Scale (SDS) [29], and perceived social support was assessed with the Multidimensional Scale of Perceived Social Support (MSPSS) [30].

### Statistical analysis

Participants were classified as low suicidality (score ≤5 on the MINI suicidality module) or high suicidality (score ≥6 on the MINI suicidality module). Country, religion, age group, sex, marital status, work status, and education were compared across low and high suicidality groups using Pearson’s chi-square tests. Age, age at first onset, length of illness, the number of past hospitalizations, MADRS score, GSI score, FSS score, SF-36’s total and subscales (bodily pain, emotional wellbeing, general health, role limitation due to emotional health, role limitation due to physical functioning, social functioning, vitality) score, SDS’s total and subscales (work and school, social and leisure, family life) score, and MSPSS’s total and subscales (family, friends, significant others) scores for low- and high-suicidality groups were compared using two-tailed Student’s t-tests for normally distributed variables and Mann–Whitney U-tests for non-normally distributed variables. A stratified logistic regression model was used to investigate predictors of high risk of suicide after controlling for age, sex, years of education, religion, work status, and total MADRS, GSI, FSS, and MSPSS scores. To account for collinearity, the country was not included as an independent variable but as a stratum in the stratified logistic regression model, because its association with other variables such as religion and educational background were high. Independent variables that were analyzed included age, sex, education, religion, work status, history of hospitalization, total MADRS score, GSI of SCL-90-R score, total FSS score, and total MSPSS score. Variables significant (p < 0.1) on univariate analysis were selected for inclusion in the multivariable model.

The null hypothesis was rejected at p < 0.05. The Statistical Package for the Social Sciences (SPSS) software, version 12.0, and SAS (version 9.3, Cary, NC) were used for all analyses.

## Results

A total of 2,023 outpatients were screened for eligibility, and 637 (31.5%) were eligible. Of the 637 outpatients that were eligible, 556 were enrolled in the study. The remaining 81 outpatients were not enrolled for the following reasons: 1) refusal/unwillingness to cooperate (n = 58); 2) insufficient patience to be interviewed (n = 14); or 3) insufficient time to participate (n = 9). All participants were compensated for their time. The mean (SD) time taken for completion of the self-administered questionnaires was 35.8 (14.1) min, and for face-to-face interview was 38.1 (13.8) min. After the interviews, nine participants were excluded from further analysis because they had no MDD. The remaining 547 participants were included in the analysis. 125 (22.9%) were classed as high suicidality (score ≥6 on the MINI suicidality module) and 422 (77.1%) were classed as low suicidality (score ≤5 on the MINI suicidality module).

### Univariate analysis of sociodemographic and clinical factors

There were significant differences in country (*χ*^2^ = 45.62, p < 0.001), religion (*χ*^2^ = 12.57, p = 0.028), sex (*χ*^2^ = 4.13, p = 0.044), work status (*χ*^2^ = 13.42, p = 0.001), and number of hospitalizations (t = 2.44, p = 0.016) between low and high suicidality groups (Table 1). The highest proportion of patients classed as high suicidality occurred in South Korea (42.6%), followed by Taiwan (31.3%), China (21.9%), and Singapore (17.5%). There were no significant differences in age, marital status, education, age at first onset, and length of illness between low and high suicidality groups (Table 1). The high-suicidality group had higher MADRS (t = 7.33, p < 0.001), GSI (t = 5.40, p < 0.001), FSS (Z = -3.191, p < 0.001), and SDS scores (t = 3.34, p = 0.001) and lower SF-36 (t = 5.09, p < 0.001) and MSPSS scores (t = 3.97, p < 0.001) than did the low-suicidality group (Table 2).

**Table 1:** Sociodemographic characteristics of major depressive disorder patients with low and high suicidality

	**Low suicidality ( *n * = 422)**		**High suicidality ( *n * = 125)**		**Comparison of low and high suicidality groups**		
	***n***	**%**	***n***	**%**	***χ***^***2***^	***df***	***p *** **value**
Country							
China	89	78.1	25	21.9	45.62	5	<0.001*
South Korea	58	57.4	43	42.6			
Malaysia	82	91.1	8	8.9			
Singapore	33	82.5	7	17.5			
Thailand	92	89.3	11	10.7			
Taiwan	68	68.7	31	31.3			
Age group							
18–29 years	128	78.5	35	21.5	4.40	4	0.355*
30–39 years	86	71.7	34	28.3			
40–49 years	76	74.5	26	25.5			
50–59 years	103	81.1	24	18.9			
60–65 years	29	82.9	6	17.1			
Sex							
Male	160	82.1	35	17.9	4.13	1	0.044*
Female	262	74.4	90	25.6			
Marital status							
Married or co-habiting	254	73.8	64	26.3	2.90	2	0.235
Widowed or divorced	50	79.9	18	20.1			
Never married	118	73.5	42	26.5			
Work status							
Employed	216	84.0	41	16.0	13.42	2	0.001*
Homemaker or student	132	72.1	51	27.9			
Unemployed	74	69.2	33	30.8			
Education							
None or primary	67	83.8	13	16.3	3.30	2	0.192
Secondary or vocational	236	74.7	80	25.3			
College	119	78.8	32	21.2			
Past hospitalization					13.76	1	.001**
None	394	93.8	104	83.2			
Presence	26	6.2	21	16.8			
Religion							
No religion	159	73.3	58	26.7	12.57	5	0.028*
Buddhist	152	v	39	20.4			
Christian	50	69.4	22	30.6			
Hindu	20	95.2	1	4.8			
Muslim	34	89.5	4	10.5			
Other	7	87.5	1	12.5			
	Mean (SD)		Mean (SD)		t	df	p value
Age (years)	39.9 (13.4)		38.7 (12.6)		0.89	545	0.376
Age at first onset (years)	36.9 (13.4)		34.8 (12.8)		1.56	544	0.119
Length of illness (weeks)	76.1 (159.3)		90.2 (174.8)		0.45	545	0.398

*P < 0.05.

**P < 0.01.

**Table 2:** Clinical characteristics of major depressive disorder patients with low and high suicidality

	**Low suicidality ( *n * = 422)**	**High suicidality ( *n * = 125)**	**Comparison of low and high suicidality groups**		
			***t *** **or *Z***	***df***	***p *** **value**
MADRS score	27.78 (7.96)	33.58 (7.14)	7.33	545	<0.001**
GSI (of SCL-90-R) score	0.13 (0.06)	0.17(0.07)	5.40	543	<0.001**
FSS score	4.92 (1.47)	5.42 (1.22)	-3.19	236.19	0.001**
SF-36 score	376.7 (124.1)	311.9 (126.8)	5.09	542	<0.001**
Bodily pain†	59.19 (27.22)	55.94 (29.46)	-1.04	543	.294
Emotional wellbeing	33.52 (18.15)	24.29 (16.89)	5.05	544	<0.001**
General health	37.15 (20.42)	32.70 (23.06)	2.07	544	0.039*
Role limitation due to emotional health†	41.45 (26.77)	30.17 (24.95)	-4.27	543	<0.001**
Role limitations due to hysical health†	50.67 (28.73)	42.85 (28.17)	-2.70	544	<0.001**
Physical functioning	79.83 (22.37)	69.82 (25.44)	-4.14	180.48	<0.001**
Social functioning	46.77 (24.87)	40.02 (25.90)	-2.63	544	0.008*
Vitality	28.41 (19.21)	16.63 (15.65)	-6.27	242.14	<0.001**
SDS score	16.52 (8.08)	19.22 (7.73)	3.34	545	0.001**
Work/school	6.23 (2.29)	7.34 (2.55)	-3.46	203.59	0.001**
Social/leisure	5.64 (3.00)	6.52 (2.84)	-2.92	544	0.003**
Family life	5.24 (3.20)	6.35 (2.95)	-3.40	543	0.001**
MSPSS score	4.56 (1.40)	3.99 (1.42)	3.97	542	<0.001**
Family	4.89 (1.65)	4.18 (1.87)	-3.64	180.60	<0.001**
Friends	4.35 (1.59)	3.85 (1.56)	3.07	542	0.002**
Significant others	4.71 (1.82)	4.18 (1.82)	-2.93	542	0.003**

†Reversely-coded variables. Higher score indicates better quality of life.

MADRS, Montgomery-Åsberg Depression Rating Scale; Global Severity Index(SCL-90-R, Symptoms Checklist Questionnaire-90-Revised); FSS, Fatigue Severity Scale; SF-36, Medical Outcome Survey Short Form-36; SDS, Sheehan Disability Scale; MSPSS, Multidimensional Scale of Perceived Social Support.

*P < 0.05.

**P < 0.01.

### Multivariate analysis for high suicidality

In the logistic regression model religion, work status, MADRS score, and FSS scores were related to high suicidality in MDD patients (Table 3). Patients who were unemployed (adjusted odds ratio (OR) 2.50, 95% confidence interval (CI) 1.27-4.90), patient who had history of hospitalization(adjusted odds ratio (OR) 2.96, 95% confidence interval (CI) 1.41-6.20), patients who had high MADRS score (adjusted OR 1.11, 95% CI 1.07-1.15), and patients who had a high FSS score (crude OR 1.36, 95% CI 1.15–1.61) had increased odds of being in the high suicidality group. The MSPSS score (adjusted OR 0.83, 95% CI 0.70–0.98) was inversely associated with high suicidality. Age, sex, years of education, religion, and marital status were not significant in the model (Table 3).

**Table 3:** **Stratified logistic regression model for high suicidality** (**stratum**: **country**)

**Values**	**Crude OR (95% CI)**	**Wald**	**p value**	**Adjusted OR† (95% CI)**	**Wald**	**p value**
Age	0.98 (0.97–1.00)	3.390	0.066	0.99 (0.97–1.01)	1.783	0.182
Sex: Female	1.55 (0.98–2.45)	3.552	0.059	1.36 (0.80–2.30)	1.304	0.254
Education						
None or primary	1	2.704	0.259			
Secondary or vocational	1.78 (0.89–3.55)	2.651	0.104			
College	1.72 (0.81–3.69)	1.976	0.160			
Marital status						
Married or co-habiting	1	3.487	0.175			
Widowed or divorced	1.29 (0.68–2.45)	0.628	0.428			
Never married	1.56 (0.97–2.50)	3.406	0.065			
Religion						
No religion	1	3.259	0.660			
Buddhist	1.39 (0.75–2.56)	1.099	0.294			
Christian	1.19 (0.61–2.36)	0.262	0.609			
Hindu	0.46 (0.05–3.91)	0.511	0.475			
Muslim	1.23 (0.33–4.60)	0.097	0.755			
Others	0.35 (0.04–2.99)	0.929	0.335			
Work status						
Employed	1	6.565	0.038	1	7.397	0.025
Homemaker or student	1.60 (0.96–2.66)	3.309	0.069	1.22 (0.69–2.16)	0.452	0.502
Unemployed	2.03 (1.15–3.59)	5.920	0.015	2.54 (1.29–5.01)	7.247	0.007
Past hospitalization						
None	1					
Presence	3.39 (1.76–6.54)	13.305	<0.001	2.90 (1.38–6.07)	7.948	0.005
MADRS score	1.13 (1.09–1.17)	51.005	<0.001	1.10 (1.06–1.14)	22.354	<0.001
GSI (of SCL-90-R) score	1.11 (1.07–1.15)	30.439	<0.001	1.04 (1.00–1.09)	3.269	0.071
FSS score	1.36 (1.15–1.61)	12.827	<0.001	1.09 (0.90–1.32)	0.813	0.367
MSPSS score	0.78 (0.67–0.91)	9.993	0.002	0.84 (0.71–1.00)	3.748	0.053

OR, odds ratio; CI, confidence interval; MADRS, Montgomery-Åsberg Depression Rating Scale; Global Severity Index (SCL-90-R, Symptoms Checklist Questionnaire-90-Revised excluding the depression subscale); FSS, Fatigue Severity Scale; MSPSS, Multidimensional Scale of Perceived Social Support.

*P < 0.05.

**P < 0.01.

†Adjusted variables: *p* < 0.1 in univariate analysis.

## Discussion

In the current study, MDD patients were categorized as low or high suicidality according to their score on the MINI suicidality module. Country, religion, sex, work status, depression severity (measured using MADRS), and the number of past hospitalizations differed between patients with low and high suicidality, but age, marital status, education level, age at first onset of MDD, and length of illness were similar in the two groups. In Malaysia and Thailand, about 10% of MDD patients were classed as high suicidality, whereas in South Korea over 40% of MDD patients were classed as high suicidality. This is consistent with recent epidemiological studies on national suicide rates. The World Health Organization reported that suicide rates in East Asian countries such as South Korea and China were much higher than in Malaysia and Thailand [3], and the prevalence of suicidal behavior has consistently been reported to be high in South Korea [31]. Differences in the suicide rates in Asian countries are related to various factors including climate, religion, financial status, and availability of suicide methods [2]. For instance, South Korea and China experience more drastic weather changes than Thailand or Malaysia, and such changes may contribute to the high suicide rate [32]. Furthermore, the abrupt social changes and economic recession in East Asian countries is likely to have influenced the suicide rate.

In the present study, there was a significant relationship between religion and suicidality. Patients who were Hindu or Muslim had a lower suicidality, which was shown to be consistent with previous reports that practicing a religion that forbids suicide, such as Islam, contributes to low suicide rates [2,5]. However, the relationship between religion and suicidality was not significant after stratifying the effect of country; thus, no independent effect of religion on suicidality was evident. There was a higher proportion of females than males in the high suicidality group [33-36]. Previous studies have revealed higher suicidality in females in Asian countries than in females in countries such as the United States and Australia [33-36]. This difference could be related to the low socioeconomic status of women, presence of abusive family relationships, and the frequent use of violent suicide methods in Asian countries [33-36]. Unemployed persons had a 2.5 times higher risk of being in the high suicidality group than employed persons. This indicates a need for social structural efforts to improve employment stability in addition to clinical interventions to lower the suicide rate [37].

The high suicidality group had more severe depression, indicated by higher MADRS scores, than the low suicidality group, and reported a greater number of psychiatric. This corresponds with existing arguments that depression severity and other comorbid conditions are crucial risk factors for suicide [9-12,18]. Further, perceived social support, assessed using the MSPSS, served as a protective factor for suicidality. According to the present results, patients who perceived a low level of support from family, friends, and significant others had a higher risk of being in the high suicidality group. The importance of social support for suicide prevention has been suggested many times in previous studies [9,11,38], and may be particularly important in the family-oriented Asian culture, where individuals with mental illness have a tendency to be hidden and isolated from society because the stigma of mental illness affects the entire family [39]. Social support should therefore be regarded as an important factor for preventing suicide, and interventions based on social relationships should be expanded in Asian countries. It is interesting that although perceived social support was a protective factor for suicidality, marital status had no significant influence on suicidality. Existing research has shown that marital status or the presence of a partner is not a protective factor for suicide in Asian countries because of the characteristics of the family system in Asian countries [2,40]. Many Asians tend to stay married due to gender inequalities and the negative perception of divorce in Asian society, but stressful martial relationships may worsen depression or increase the suicidality [2,40].

Age [9], education [13], and sex, which have all been found to be risk factors in previous studies, were not significantly related to suicidality in the logistic regression. This inconsistency may be due to the differences in subject characteristics, ethnicities and assessment tools. Age effects could not be detected as they are not linear within the range of age reported.

It is possible that suicidality was underestimated due to bias in self-reports, as patients may be embarrassed to admit suicidal behavior and mental problems. Additionally, the samples may not have been representative of each country as a whole, as they comprised clinical samples drawn from tertiary care centers. Recruitment was biased toward MDD patients who used health care institutions, and there may be differences in health care systems among the six countries that participated in the study. Also, This study was cross-sectional in design, making it impossible to identify a casual relationship between the identified risk factors and suicidality. Specific risk factors that contributed to the national differences in suicidality risk among MDD patients were not examined. Finally, while impact of country and religion were investigated in the present study, influence of ethnicity was not explored due to homogeneity in terms of ethnicity in most countries. A recent epidemiological study in Malaysia (n = 20,552) by Maniam et al. (2013) showed that suicidal ideation was significantly associated with Indian ethnicity (especially among those who were Hindu) compared with Malays and Chinese [41,42]. Further study about the influence of ethnicity on suicidality may be needed in the clinical as well as the general population. Despite these limitations, the present study revealed that a variety of sociodemographic and clinical factors were associated with high suicidality in MDD patients from six Asian countries. In particular, as with severity of MDD, non-clinical features such as social support from various sources were found to be associated with suicidality. This association with cultural and social factors may explain the limited relationship between MDD rate and suicidality in Asian countries. Further, identification of these factors may facilitate the identification of MDD patients at risk of suicide and the provision of suicide prevention guidelines.

## Conclusion

It is well-known that Asian countries have high suicide rates. In addition, the profiles of risk and protective factors of suicide in Asian countries may differ from those of Western countries. However, comprehensive investigation of the characteristics of suicide in the countries was relatively few. This study aimed to examine the sociodemographic and clinical factors associated with suicidality in MDD patients from six Asian countries. The high suicidality group was found to have higher depressive symptoms, general psychopathology and disability scores and lower quality of life and social support scores than the low suicidality group. Moreover, some religion, unemployment and past psychiatric hospitalization were associated with high suicidality in MDD patients in Asian countries. These findings point to the need for a careful evaluation of the risk factors for the suicidality in Asian countries. These factors may facilitate the identification of MDD patients at risk of suicide.

## Competing interests

The authors declare that they have no competing interests.

## Authors’ contributions

AL and AL participated in data analysis and drafted the manuscript. CL and DB carried out data analysis. All other authors participated in the design of the study and collected raw data. All authors read and approved the final manuscript.

## Pre-publication history

The pre-publication history for this paper can be accessed here:

http://www.biomedcentral.com/1471-244X/14/37/prepub
